# Evaluation of reference genes for reverse transcription quantitative real-time PCR (RT-qPCR) studies in *Silene vulgaris* considering the method of cDNA preparation

**DOI:** 10.1371/journal.pone.0183470

**Published:** 2017-08-17

**Authors:** Pavla Koloušková, James D. Stone, Helena Štorchová

**Affiliations:** Plant Reproduction Laboratory, Institute of Experimental Botany v.v.i., Academy of Sciences of the Czech Republic, Prague, Czech Republic; University of Naples Federico II, ITALY

## Abstract

Accurate gene expression measurements are essential in studies of both crop and wild plants. Reverse transcription quantitative real-time PCR (RT-qPCR) has become a preferred tool for gene expression estimation. A selection of suitable reference genes for the normalization of transcript levels is an essential prerequisite of accurate RT-qPCR results. We evaluated the expression stability of eight candidate reference genes across roots, leaves, flower buds and pollen of *Silene vulgaris* (bladder campion), a model plant for the study of gynodioecy. As random priming of cDNA is recommended for the study of organellar transcripts and poly(A) selection is indicated for nuclear transcripts, we estimated gene expression with both random-primed and oligo(dT)-primed cDNA. Accordingly, we determined reference genes that perform well with oligo(dT)- and random-primed cDNA, making it possible to estimate levels of nucleus-derived transcripts in the same cDNA samples as used for organellar transcripts, a key benefit in studies of cyto-nuclear interactions. Gene expression variance was estimated by RefFinder, which integrates four different analytical tools. The *SvACT* and *SvGAPDH* genes were the most stable candidates across various organs of *S*. *vulgaris*, regardless of whether pollen was included or not.

## Introduction

The quantification of gene expression based on RT-qPCR is an increasingly important method across diverse fields of plant biology. Initially developed in *Arabidopsis* [1] and crop plants [2, 3], it has been adapted for use in wild species investigated by ecologists and population geneticists [4, 5]. The accuracy of RT-qPCR results is influenced by numerous factors including the quality of RNA, method of cDNA preparation, PCR efficiency, and very importantly, by the choice of reference gene [6]. An ideal reference gene has to show invariant expression across a range of tissues, developmental stages and environmental conditions. However, expression of the vast majority of genes fluctuates in response to various stresses [7, 8, 9, 10, 11, 12, 13, 14, 15], circadian rhythms [12, 16], or by developmental stage [10, 17, 18, 19, 20, 21]. Furthermore, most genes show substantial differences between gametophyte and sporophyte [20, 22]. It is therefore necessary to confirm invariant expression of a candidate reference gene in relevant tissues, organs and under experimental treatments before it is used in an experiment. A reference gene successfully used in one species may serve as a starting point for identifying them in another, but cannot necessarily be applied in a different, albeit related, species. The choice of reference gene(s) should be always justified for the given species and conditions as recommended by MIQE guidelines (*M*inimum *I*nformation for Publication of *Q*uantitative Real-time PCR *E*xperiments) [6].

Although the majority of angiosperms are hermaphroditic, some species produce female, male or hermaphroditic flowers on the same individual (monoecy), or on distinct individuals (dioecy). In these species, gender-specific expression patterns, in addition to organ- and tissue-specificity, has to be considered, when evaluating reference genes [4, 23, 24].

*Silene vulgaris* has emerged as a model system for investigating gynodioecy, a plant breeding system characterized by the co-occurrence of females (F) and hermaphrodites (H) [25]. The female gender is attributable to the interaction of cytoplasmic male sterility (CMS) genes, located in the mitochondrial genome, and nuclear genes [26]. This system represents a textbook example of cyto-nuclear interaction [27].

Recent publications of the complete mitochondrial genomes [28] and transcriptomes of *S*. *vulgaris* [29, 30] pave the way for detailed investigations of gene expression and function in this species. RT-qPCR plays an essential role in gene expression studies, but no detailed examination of reference genes suitable for transcript level normalization across various tissues and organs of *S*. *vulgaris* has been published. This study aims to identify reference genes with invariant expression in F and H plants of *S*. *vulgaris*, across leaves, roots, flower buds and pollen.

To investigate the role of gene expression in cyto-nuclear interactions, it is important to estimate both organellar and nucleus-derived transcript levels in the same cDNA specimens. Unlike cytoplasmic mRNAs, organellar mRNAs cannot be efficiently reverse transcribed using oligo(dT)-primers, because they often lack poly(A) tails [31, 32]. To achieve balanced reverse transcription of both cytoplasmic and organellar RNAs, cDNA synthesis primed with random oligomers is necessary. On the other hand, most gene expression studies rely on oligo(dT)-priming, taking an advantage of selective enrichment of poly(A)-tailed mRNAs. To better study the transcription of genes involved in cyto-nuclear interactions in *S*. *vulgaris*, reference genes performing well with both of these cDNA priming methods will be identified. This is the first study that reported the comparison of two different cDNA synthesis method on the transcription stability of reference genes. Accordingly, we have measured the stability of expression of eight reference gene candidates in *S*. *vulgaris* with cDNAs generated by both oligo(dT)- and random-priming.

Finally, we demonstrated the utility of the best candidate reference genes by normalizing the expression of the nuclear *MutS HOMOLOGUE 1* (*MSH1*) gene, encoding the mitochondrial- and plastid-targeted protein which influences recombination and replication in plant organelles [33, 34].

## Material and methods

### Plant material

*S*. *vulgaris* plants were grown from seedlings to mature plants in 1.5 L pots filled with a substrate of coconut coir, Vermiculite and Agroperlite (1:1:1 ratio) in the IEB greenhouse under a 16 hour light and 8 hour darkness period, temperature between 18 and 24°C and humidity around 80%. Plants were regularly watered and fertilized with a combination of Kristalon fertilizers (Agro CS, Česká Skalice, Czech Republic) and Ca(NO_3_)_2_·2H_2_O (0.66 g of Kristalon-Fruit and flower, 0.8 g of Kristalon-Autumn and 0.53 g of Ca(NO_3_)_2_·2H_2_O per L). Temperature, humidity and light conditions varied slightly with outside conditions due to the greenhouse’s semi-insulated design. We used 4 H plants for the dataset with pollen, and 3 F and 3 H plants for the other datasets.

### RNA extraction and cDNA synthesis

Tissue samples were taken from mature plants (flower buds about 0.5 cm long, young upper leaves, mature pollen after anther dehiscence and apical roots). A maximum of 100 mg tissue sample was snap-frozen in liquid nitrogen and stored at -80°C until further use. Plant material was homogenized with mortar and pestle in liquid nitrogen, total RNA was extracted using the RNeasy Plant Mini kit (Qiagen, Valencia, USA) and treated by DNaseI (Ambion, Foster City, USA). To perform the non-RT control, treated RNA was used as a PCR template with specific *S*. *vulgaris* primers (2 min at 94°C initial denaturation followed by 36 cycles: 1 min at 93°C, 1 min at 58°C and 1 min s at 72°C). This produced no amplification, confirming the absence of DNA contamination.

RNA was visualised on 0.8% agarose gel under RNase-free conditions. The concentration of RNA was quantified using a NanoDrop ND-1000 spectrophotometer (NanoDrop Technologies, Wilmington, DE, USA). RNA samples with A260/A280 ratios from 1.9 to 2.1 and A260/A230 ratios between 2.0 and 2.2 were used for cDNA synthesis. An equal amount of total RNA (500 ng) from each tissue sample was reverse transcribed with Transcriptor High Fidelity cDNA synthesis kit (Roche Applied Science, Mannheim, Germany) using oligo(dT)- or random hexamer-primers, in a 20 μL reaction volume according to the manufacturer’s instructions. The reverse transcription reaction was subsequently diluted to 20x with RNase-free water for RT-qPCR.

### Selection of candidate reference genes and primer design

Twenty candidate reference genes were selected based on their previous applications in plant gene expression studies. They included *18S rRNA* [35, 36, 37, 38], *ACTIN* (*ACT*) [38, 39, 40], *ATP-BINDING CASSETTE I6* (*ABCI6*) [12], *CLATHRIN ADAPTOR PROTEIN-2* (*AP2M*) [2], *CONSERVED OLIGOMERIC GOLGI COMPLEX* (*COG*) [41], *CYCLOPHILIN* (*CYP*) [42, 43, 44], *ELONGATION FACTOR 1 alpha* (*ELF1α*) [38, 44, 45, 46, 47], *GLYCERALDEHYDE-3-PHOSPHATE DEHYDROGENASE* (*GAPDH*) [48, 49, 50], *S-ADENOSYLMETHIONINE DECARBOXYLASE* (*SAMDC*) [51], *SAND family protein* (*SAND*) [2, 50], *SKP1/ASK-INTERACTING PROTEIN 16* (*SKIP16*) [52], *TIP41-like family protein* (*TIP41*) [52], A*LPHA TUBULIN* (*TUBA*) [9, 53], *BETA TUBULIN* (*TUBB*), *UBIQUITIN-CONJUGATING ENZYME 18* (*UBC18*) [51], *UBIQUITIN 4* (*UBQ4*) [54], *UBIQUITIN 5* (*UBQ5*) [55], *UBIQUITIN 6* (*UBQ6*) [56], *POLYUBIQUITIN 14* (*UBQ14*) [54], and *VACUOLAR MEMBRANE ATPASE 10* (*VMA10*) [41]. All amino acid sequences were blasted against the *S*. *vulgaris* transcriptome database (http://silenegenomics.biology.virginia.edu/) using blastx with a cutoff of e≤0.001, identifying isotigs with nearly complete coding sequences and overall amino acid similarity between *Arabidopsis* and *S*. *vulgaris* > 60% (Table 1). These isotig sequences from *S*. *vulgaris* were selected for primer development. At least two primer pairs were designed for each isotig using Primer 3 (http://simgene.com/Primer3) as follows: length of primers 20–24 nt, size of amplicons 90–200 bp; the function penalty weight for primer pairs, of any complementarity, was set to 1.0. Similarly, the sequence of *SvMSH1* was retrieved from the *S*. *vulgaris* transcriptome database by a blastn search of sugar beet *MSH1* mRNA (XM010692765). The final *SvMSH1* sequence (total length 4007 bp, ORF 3414 bp) was assembled from 5 isotigs, with gaps filled by PCR and Sanger sequencing.

**Table 1:** Description of *Silene vulgaris* candidate reference genes.

*S*. *vulgaris*		Arabidopsis		
Gene Symbol	Isotig*	Ortholog Locus	Gene Product	Amino Acid Identity between Arabidopsis and *S*. *vulgaris* (%)
*SvELF*	05995	AT1G07920	Elongation factor 1A	94
*SvGAPDH*	26878	AT3G04120	Glyceraldehyde-3-phospate dehydrogenase	91
*SvTUBA*	05561	AT1G04820	Alpha Tubulin	96
*SvTUBB*	07210	AT5G62700	Beta Tubulin	95
*SvACT*	09472	AT5G09810	Actin	96
*SvCOG*	34935	AT4G24840	Golgi (COG) Complex Component	63
*SvCYP*	11915	AT4G38740	Cyclophylin ROC1	83
*18S rRNA*	36580	AT3G41768	18S ribosomal RNA	89

* *S*. *vulgaris* transcriptomic database available at http://silenegenomics.biology.virginia.edu

### Quantitative real-time PCR (qPCR)

qPCR was carried out in 96-well plates on a LightCycler^®^ 480 Instrument II (Roche Molecular System, Germany) using LightCycler^®^ 480 SYBR Green I Master (Roche Applied Science, Mannheim, Germany). One sample reaction was run in four replicates (two replicates per plate, to check run-to-run variation) in 10 μL total volume containing 5 μL of 2x SYBR Green I Master mix, 2.5 μL of diluted cDNA and gene-specific primers in proper concentrations (Table 2).

**Table 2:** Details of qPCR primers and amplicons.

Gene Symbol	Primer Pair	Primer Sequence Forward / Reverse	Ann Temp (°C)	Optimized Primer Concentration (pM)	cDNA Amplicon Length (bp)	qPCR efficiency
*SvELF*	Sv_ELF 1	TCTCCCTGGTGACAATGTTGGT / GAACTGGGGCATATCCGTTACC	60	0.5	176	1.0
	Sv_ELF 2	GCATGCACTTCTTGCTTTCA / GACCTTGTCGGGATTGTACC	60	0.5	160	1.0
*SvGAPDH*	Sv_GAPDH 1	GGCCAAGGTTATCAATGACAG / CCTTCCACCTCTCCAGTCCT	60	0.4	119	1.0
	Sv_GAPDH 2	GATCGGAATCAACGGATTTG / TGGACCATGAACACTGTCGT	60	0.5	151	0.98
*SvTUBA*	Sv_TUB A	ACATGGCTTGCTGTCTGATG / TGGGGGCTGGTAGTTGATAC	60	0.4	146	1.0
*SvTUBB*	Sv_TUB B	ACAACCCATCGACTCGAAAC / GCAACATGAATGACCTCGTG	60	0.5	173	1.0
*SvACT*	Sv_ACT	GGGCTGTGATCTCTTTGCTC / ATTGTTCGGTATGGAAGCTC	60	0.4	163	0.96
*SvCOG*	Sv_COG	CCTGTTCGCCATTCTCCTTA / CGACTTCAGATGCCAATTCA	60	0.5	166	1.0
*SvCYP*	Sv_CYP	TCGCAGTTCTTCATCTGCAC / AGCCTTCACAACATCCAACC	60	0.4	96	1.0
*18S rRNA*	Sv_18S rRNA	CCTCCAATGGATCCTCGTTA / AAACGGCTACCACATCCAAG	63	0.3	154	1.0
*SvMSH1*	Sv_MSH_1	CTGAGGATTTCCTCCCCA/ ACCAAACTGCTTCGTGTC	60	0.5	105	1.0

**Fig 1:**
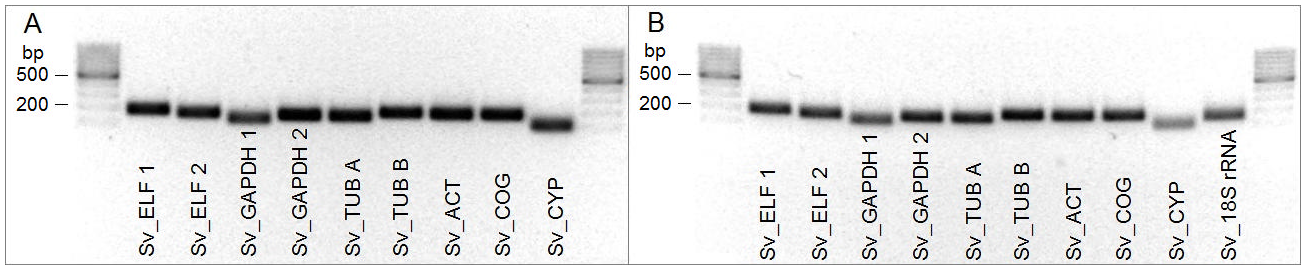
qPCR amplicons generated with cDNA from leaf tissue by the indicated primer pairs. Agarose gel (1.5%) with specific qPCR products of expected sizes. **A**: oligo(dT)- primed amplicons; **B**: random hexamer-primed amplicons. Size standards are loaded on the left and right sides—GeneRuler 100 bp DNA Ladder.

Cycling conditions: 5 min at 95°C for initial denaturation followed by 50 cycles: 10 s at 95°C, 10 s at 60°C and 15 s at 72°C, except for 18S rRNA primers where annealing was set to 10 s at 63°C. The PCR efficiency for each assay was calculated with LightCycler^®^ 480 software from the calibration curve based on a serial dilution of cDNA (most often 20x, 40x, 80x, 160x, 320x). Specificity of qPCR in each sample was confirmed by melting curve analyses. The qPCR amplicons (Fig 1) were sequenced to confirm their identity.

### Validation of gene expression using RNA-seq data

RNA-seq data obtained by Illumina HiSeq (single reads, 50 cycles) from eight cDNA libraries constructed by oligo(dT)-priming from the flower buds and leaves of H and F plants were used to evaluate the expression variance of each reference gene candidate. Amplicon coverage was estimated as follows: each primer sequence, as well as the 30 nt immediately upstream and downstream were used for read alignment, ensuring complete coverage of the target primer sequence. The sequences between primer pairs would have attracted reads derived from alternative transcripts and, therefore, not unique to the focal primer pairs and were not used for read alignment. Thus, the reference sequence for each primer pair was 160 mappable bases (two 20 nt primer sequences and four flanking 30 nt regions). Occasionally, polymorphism in the flanking regions necessitated multiple reference sequences per primer pair to capture all the reads derived from RNA potentially amplified by a particular primer pair. Reads from individual transcriptomes were aligned to these reference sequences using Bowtie2 [57], allowing zero mismatches and requiring that the full read length aligned. The average coverage of each sequence was obtained by BEDTools genomecov tool [58]. The coverage values were multiplied by 10e9, divided by sequence length adjusted for edge effects (50% average coverage in flanking regions gives an adjusted length of 100 nt rather than 160 nt), divided by 50 (read length), and lastly divided by the total number of reads from each sample. If multiple mapping sequences were needed for a particular primer pair, their coverage values were summed. The resulting number is equivalent to RPKM (reads per 1 thousand bp of sequence length and 1 million total reads). Coefficient of variation (CV) was calculated as the standard deviation across samples divided by the mean value and expressed as a percentage.

### Statistical evaluation

The web tool RefFinder [59] (http://fulxie.0fees.us/) which integrates four widely used algorithms: geNorm [60], NormFinder [61], BestKeeper [62] and the comparative delta-Ct method [63] was used to estimate the stability of candidate reference genes, providing us comprehensive information for ranking the candidate reference genes. We used all four technical cycle threshold values (C_t_) replicates as input when calculating stabilities. Expression stability was also tested using the stand-alone programs geNorm and NormFinder, where relative quantities (RQ) calculated with qPCR efficiencies, rather than raw C_t_ data, were used as inputs, as directed in the geNorm manual.

*MSH1* expression data were analyzed by a two-way analysis of variance (ANOVA) with gender and organ modeled as fixed-effects. Differences between means were tested using Tukey’s test and a critical value of *P* < 0.05. Analyses were performed using the software SPSS 15.0.

## Results

### Expression profiles of candidate reference genes

We selected eight *S*. *vulgaris* isotigs homologous to reference genes previously employed for RT-qPCR normalization in plants (Table 1): *SvELF1α* [38], *SvGAPDH* [50], *SvTUBA*, *SvTUBB*, *SvACT* [52], *SvCOG* [41], *SvCYP* [44], and *18S rRNA* [64]. We performed several RT-qPCR assays for each gene and chose the primer pairs which worked with a PCR efficiency of at least 1.95 and produced only the specified PCR products (Table 2). We continued with two primer pairs targeting different genic regions of *SvELF1α* and *SvGAPDH* and with one primer pair for the rest of the candidates.

As we aimed to find reliable reference genes to evaluate gene expression across various tissues of both F and H plants of *S*. *vulgaris*, we examined two groups of plants. The first group, H_BLRP, contained only H individuals (4 plants), which made it possible to measure gene expression in pollen (P) in addition to flower buds (B), leaves (L) and roots (R). The second group (named HF_BLR) consisted of 3 H and 3 F plants and was used to determine gene expression in flower buds (B), leaves (L) and roots (R).

We also tested whether the same reference genes could be used with cDNAs prepared by different priming methods. Accordingly, we performed RT-qPCR measurements of all the genes except 18S rRNA in parallel with cDNA primed either with oligo dT or with random hexamers. The 18S rRNA expression was estimated only in random hexamer-primed cDNA, because their lack of poly(A) tails precluded oligo(dT)-priming.

**Fig 2:**
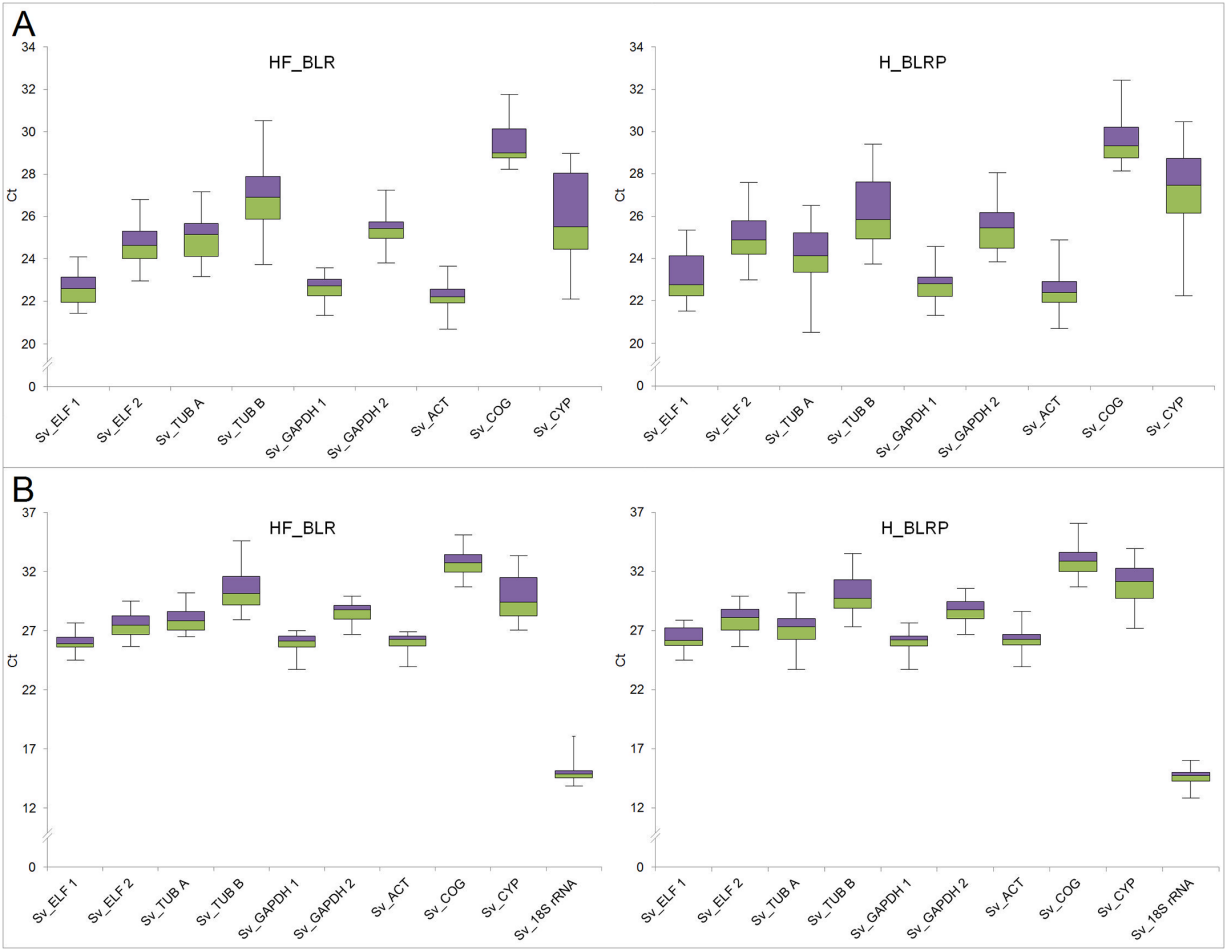
Box-plot of threshold cycle (C_t_) values of the candidate reference genes. Flower buds, leaves, roots and pollen (BLRP) or in flower buds, leaves, roots (BLR) were analyzed. **A**: Oligo dT primed cDNAs. **B**: Random hexamer-primed cDNAs. Graph shows the median values as line across the box. Lower and upper box boundaries indicate the 25^th^ percentile and the 75^th^ percentile, respectively. Whiskers represent the maximum and minumum values.

Fig 2 shows a comparison of C_t_ values of the candidate reference genes obtained with oligo dT primed or random hexamer-primed cDNA. Medians and percentiles of C_t_ values for the candidate genes are consistent between the two datasets. The medians from hexamer-primed cDNAs are several cycles higher than those obtained with oligo(dT)-primed cDNA, because hexamer-primed cDNA contains a much lower proportion of mRNA-derived reverse transcripts.

The two primer pairs designed for the *SvELF1α* and *SvGAPDH* genes differed in median C_t_ values. This may be caused by the distinct PCR efficiencies of RT-qPCR assays (in the case of Sv*GAPDH*) or by targeting multiple loci. The C_t_ values for two tissue sets with pollen (H_BLRP—buds, leaves, roots, pollen) and without pollen (HF_BLR—buds, leaves, roots) were concordant. The set with pollen exhibited larger variation.

### Stability of expression of candidate reference genes

The two most stably expressed candidate reference genes of *S*. *vulgaris* across the oligo dT primed tissue set with pollen (H_BLRP), as determined by RefFinder, were *SvGAPDH* (primer pair Sv_GAPDH 1) and *SvACT* (Table 3). They were followed by Sv*ELF1α* (primer pair Sv_ELF 1) and *SvGAPDH* (primer pair Sv_GAPDH 2). Random-primed cDNA provided a very similar order of reference gene candidates for the complete set of tissues including pollen (H_BLRP). A minor difference in the oligo(dT)-primed ranking was the exchange of first and second positions, which placed *SvACT* on the top. *18S rRNA*, frequently used to normalize random primed cDNA, was not the best performer, ranking, on average, fifth.

**Table 3:** RefFinder output. Recommended comprehensive ranking of candidate reference gene primer pairs in analyzed datasets from the most stable (1) to the least stable (9,10) genes.

rank	oligo_HF_BLR	stability	oligo_H_BLRP	stability	random_HF_BLR	stability	random_H_BLRP	stability
1	Sv_GAPDH 1	1.19	Sv_GAPDH 1	1.00	Sv_ACT	1.19	Sv_ACT	1.57
2	Sv_ACT	1.41	Sv_ACT	1.86	Sv_GAPDH 1	2.6	Sv_GAPDH 1	2.11
3	Sv_COG	3.41	Sv_ELF 1	3.13	Sv_ELF 1	2.21	Sv_ELF 1	2.45
4	Sv_GAPDH 2	3.94	Sv_GAPDH 2	4.12	Sv_GAPDH 2	3.72	Sv_GAPDH 2	3.72
5	Sv_ELF 1	4.68	Sv_ELF 2	5.00	Sv_TUB A	5.23	Sv_18S rRNA	4.30
6	Sv_TUB A	5.96	Sv_COG	5.5	Sv_ELF 2	6.24	Sv_COG	5.23
7	Sv_ELF 2	6.74	Sv_TUB B	7.24	Sv_18S rRNA	7.33	Sv_ELF 2	6.24
8	Sv_TUB B	8.00	Sv_TUB A	7.74	Sv_TUB B	7.45	Sv_TUB B	8.24
9	Sv_CYP	9.00	Sv_CYP	9.00	Sv_CYP	8.97	Sv_TUB A	8.74
					Sv_COG	9.46	Sv_CYP	10.00

*RefFinder stability values represent geometric means of ranking values

When pollen was excluded in the oligo(dT)-dataset, the two most stable candidates remained, but the third most stable gene became *SvCOG*, which ranked lower in dataset including pollen (Table 3). Its overall transcript level was the lowest of the examined candidate genes (Fig 2).

Hexamer-primed cDNA gave results consistent with the oligo(dT)-dataset, with *SvACT* and *SvGAPDH* (primer pair Sv_GAPDH 1) as the most stable reference genes. The least stable gene was *SvCYP*, regardless of the method used for cDNA priming, which agreed with its highest variation in C_t_ values (Fig 2), except the random primed HF_BLR dataset, wherein *SvCOG* was the least stable.

The stability values calculated by geNorm (S1 Fig) were in general agreement with RefFinder, although the random HF_BLR dataset was ordered slightly differently by these programs. Ranking orders generated by RefFinder and NormFinder (S1 Table) showed more differences, but they agreed in the placement of at least two candidates in the first three positions.

### Validation of reference gene candidates using RNA-seq data

To confirm the results obtained by statistical analyses of RT-qPCR estimations of gene expression values, we utilized RNA-seq data from flower bud and leaf transcriptomes obtained from 5 F and 3 H plants. We calculated read coverage of the respective amplicon regions and ordered candidate genes according to CV (Table 4). This analysis designated *SvACT* as the most stably expressed gene of our survey in concordance with RT-qPCR-based assays. This candidate reference gene was followed by *SvGAPDH* (primer pair Sv_GAPDH 1). As with the RT-qPCR results, the least stable candidate appeared to be *SvCYP*. The ranking generated from RNA-seq data agreed with the RefFinder output obtained for oligo_HF_BL dataset, which was constructed using the same cDNA priming method as used for RNA-seq. In contrast, RefFinder output based on random-primed cDNA differed from RNA-seq ranking, particularly on the stability of Sv_COG.

**Table 4:** Supporting RNA-seq analysis. Rank of analysed genes (primer pairs) according to their coefficients of variation (CV) for RPKM normalized transcript coverage. Maximum fold change (*mfc*) and sample standard deviation (*s*^*2*^) are also presented. Oligo(dT)-cDNA libraries were obtained from buds and leaves from H and F plants. RefFinder output for HF_BL is shown for the comparison.

rank	RNA-seq oligo_HF_BL	$\bar{RPKM}$	*mfc*	*s*^*2*^	CV	RefFinder oligo_HF_BL	RefFinder random_HF_BL
1	Sv_ACT	310	0.55	44	0.14	Sv_ACT	Sv_GAPDH 1
2	Sv_GAPDH 1	453	1.0	122	0.27	Sv_GAPDH 1	Sv_ACT
3	Sv_COG	4.5	2.5	1.5	0.33	Sv_COG	Sv_GAPDH 2
4	Sv_TUB B	12	3.3	4.6	0.38	Sv_GAPDH 2	Sv_ELF 1
5	Sv_GAPDH 2	61	2.7	25	0.41	Sv_ELF 1	Sv_TUB A
6	Sv_TUB A	41	4.1	25	0.61	Sv_TUB A	Sv_ELF 2
7	Sv_ELF 1	275	6.9	178	0.64	Sv_ELF 2	Sv_CYP
8	Sv_ELF 2	311	5.0	205	0.66	Sv_TUB B	Sv_TUB B
9	Sv_CYP	36	400	41	1.13	Sv_CYP	Sv_COG

### Quantification of *SvMSH1* expression

**Fig 3:**
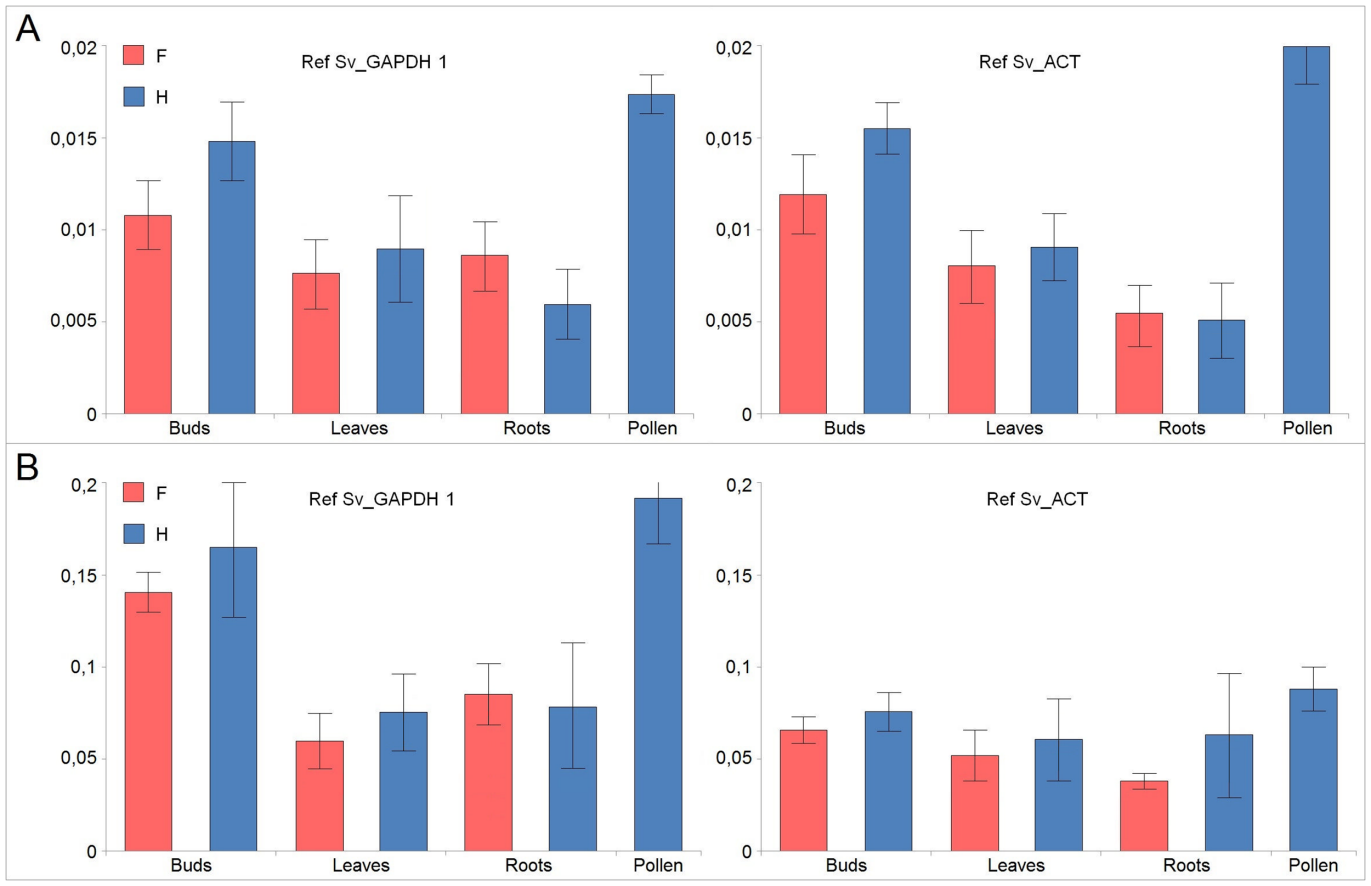
Relative expression of the *MSH 1* gene. Vegetative organs and pollen of *S*. *vulgaris* were normalized with SvGAPDH 1 or Sv_ACT primer pairs. **A**: Oligo(dT)-primed cDNAs. **B**: Random hexamer-primed cDNAs. Results from three independent measurements are presented with mean values and standard errors.

To demonstrate the utility of the selected reference genes for the normalization of gene expression across various tissues of *S*. *vulgaris*, we estimated *MSH1* transcript levels in pollen, flower buds, leaves and roots (Fig 3).

The *SvACT* and *SvGAPDH* (primer pair Sv_GAPDH 1) reference genes provided generally consistent results with both oligo(dT)- and random-primed cDNA. *SvMSH1* expression was highest in pollen and lowest in roots, where higher standard errors were reported in random-primed cDNA than in oligo(dT)-primed cDNA. Although roots were thoroughly washed before RNA extraction, trace amounts of soil might have interferred with random-primed cDNA synthesis in some samples, which could cause higher variation among the specimens.

There were no statistically significant differences between F and H plants.

## Discussion

The choice of appropriate reference genes for the normalization of transcript levels is an essential prerequisite for obtaining accurate RT-qPCR results. Candidate reference genes may be evaluated experimentally, using various statistical sofware approaches [60, 61, 62, 63]. The increasing availability of comprehensive transcriptomic data has also made *in silico* analyses of gene expression variation possible, as was recently demonstrated in *Silene latifolia* [4]. We also used read coverages calculated from leaf and flower bud transcriptomes of *S*. *vulgaris* to independently support the expression stability of candidate reference genes. Ultimately, we primarily relied on the experimental approach, because we aimed to find reference genes expressed invariantly across tissues and organs of *S*. *vulgaris*, including pollen, for which RNA-seq data were not available.

The *SvACT* and *SvGAPDH* genes were the least variable candidates across tissues and organs of *S*. *vulgaris*, regardless of whether pollen was included as determined by RefFinder (Table 3). They also exhibited the lowest CV based on transcriptomic data from leaves and flower buds (Table 4). These two genes were stably expressed with cDNAs generated by both oligo(dT)- and random-priming.

However, the method of cDNA construction appears to have affected the more variable genes—particularly *SvCOG*. This gene was the least stable candidate in random-primed cDNA from leaves, flower buds and roots, because of low *SvCOG* transcript levels. As the majority of random-primed cDNA is reverse transcribed from rRNA, the portion of mRNA-derived cDNA is much lower than in oligo(dT)-primed cDNA. Very low concentrations of *SvCOG* cDNA in the random primed samples made its RT-qPCR measurements less accurate and reproducible.

The RefFinder tool [59] which compilates four algorithms: geNorm [60], NormFinder [61], BestKeeper [62] and the comparative delta-Ct method [63] did not evaluate candidate genes congruently with standalone geNorm and NormFinder software. This discrepancy among various analytical tools has been described previously [23, 65, 66, 67, 68]. However, despite modest inconsistencies, Sv_ACT and Sv_GAPDH 1 primer pairs were consistently ranked among the first three most stable reference genes, no matter which analytical tool was applied.

Both *GAPDH* [5, 23, 48, 49, 50, 65] and *ACT* [38, 39, 40] are often used as reference genes for gene expression quantitation in angiosperms, including the genus *Silene* [4]. The *ACT* gene was reported to be affected by stress conditions [69], but our sample sets did not include stressed individuals. As the stability of reference genes has to be evaluated under specific experimental parameters [56], the selection of reference genes appropriate under stress in *S*. *vulgaris* would require further validation.

18S rRNA is sometimes utilized for expression normalization in angiosperms [70, 71], but there are also numerous reports about its expression instability [13, 14]. 18S rRNA exhibited variable transcript levels in *S*. *vulgaris* despite very high expression and cannot be recommended as a reference gene.

We used the *SvACT* and *SvGAPDH* genes to normalize *SvMSH1* transcript levels in oligo(dT)-primed and random primed cDNA. The two methods of cDNA preparation provided generally consistent results, particularly when *SvGAPDH* has been used as a reference. The *MSH1* gene not only influences organelle genome stability, but also impacts DNA methylation, flowering time, branching pattern and abiotic stress tolerance in *Arabidopsis* [72]. It will be beneficial for the study of cyto-nuclear inreactions in *S*. *vulgaris* to estimate both nucleus-encoded *MSH1* transcripts and organellar transcripts in the same cDNA specimens.

In conclusion, we recommend two reference genes, *SvACT* and *SvGAPDH*, to be applied in transcript level estimations across various organs of F and H individuals of *S*. *vulgaris*. They were selected from a broader set of candidates by an experimental approach and are suitable for random primed as well as oligo(dT)-primed cDNA.

## Supporting information

S1 Fig The outputs from geNorm depicting stability values. A. oligo(dT)-primed cDNA, B. random hexamer-primed cDNA.
(PDF)

S1 Table NormFinder outputs. The expression stability values for oligo(dT)- and random-primed cDNA.
(DOCX)
