# Supplementary material for: Evaluation of reference genes for reverse transcription quantitative real-time PCR (RT-qPCR) studies in Silene vulgaris considering the method of cDNA preparation
Source: PLoS One. 2017 Aug 17;12(8):e0183470. doi: 10.1371/journal.pone.0183470 (PMC5560574; doi:10.1371/journal.pone.0183470)

Figure S1. The outputs from GeNorm depicting stability values. A. oligo dT primed cDNA, B. random hexamer primed cDNA.

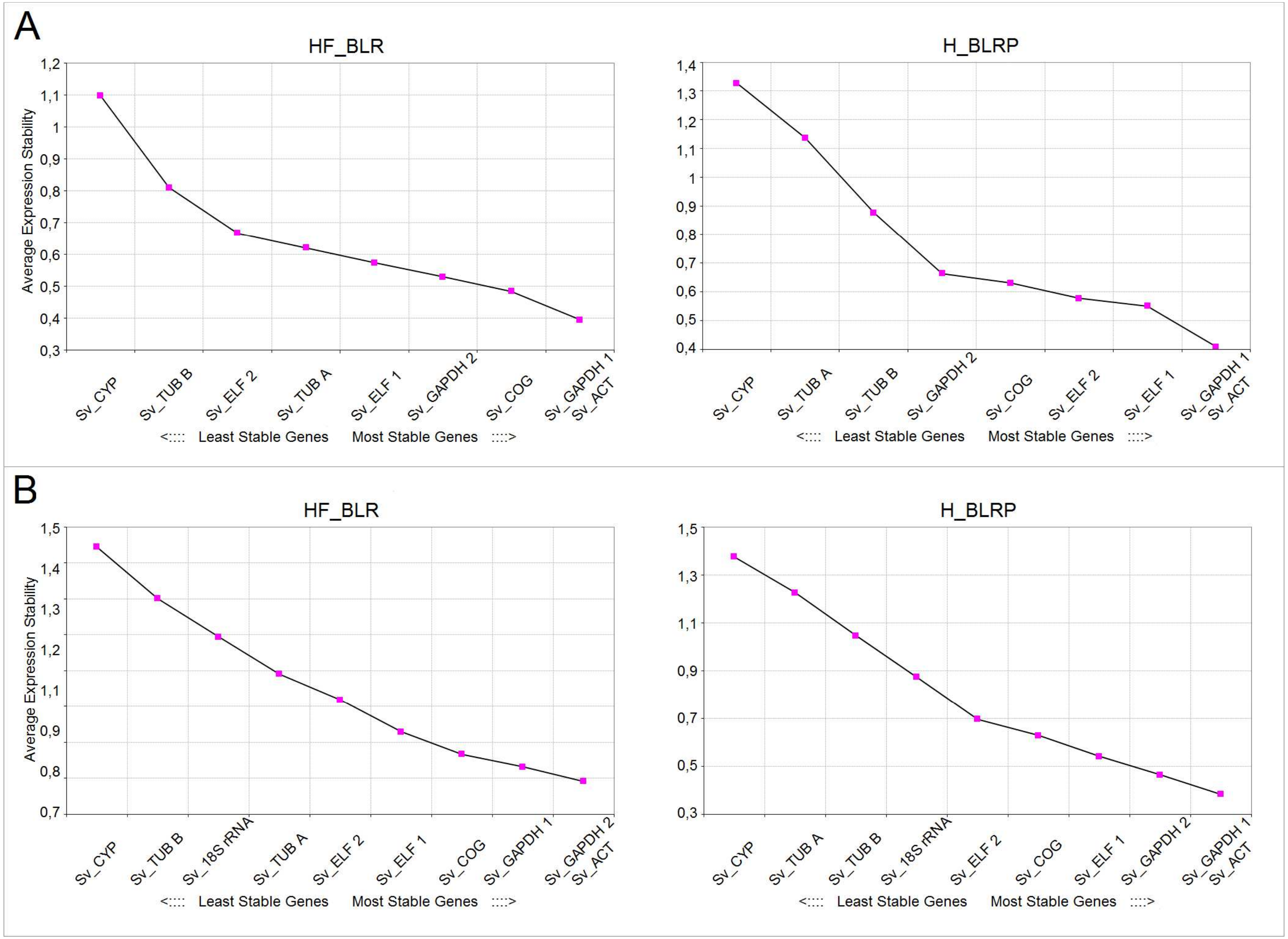

Supplement: S1 Fig — A. oligo(dT)-primed cDNA, B. random hexamer-primed cDNA. (PDF) [file pone.0183470.s001.pdf]
