# Supplementary material for: Evaluation of reference genes for reverse transcription quantitative real-time PCR (RT-qPCR) studies in Silene vulgaris considering the method of cDNA preparation
Source: PLoS One. 2017 Aug 17;12(8):e0183470. doi: 10.1371/journal.pone.0183470 (PMC5560574; doi:10.1371/journal.pone.0183470)
Supplement: S1 Table — The expression stability values for oligo(dT)- and random-primed cDNA. (DOCX) [file pone.0183470.s002.docx]

**Table S1.** NormFinder outputs showing expression stability values for oligo dT and random primed cDNA.

| **rank** | **Oligo HF_BLR** | **stability** | **Oligo H_BLRP** | **stability** | **Random HF_BLR** | **stability** | **Random H_BLRP** | **stability** |
| --- | --- | --- | --- | --- | --- | --- | --- | --- |
| 1 | Sv_ACT | 0.216 | GAPDH1 | 0.214 | Sv_ELF1 | 0.155 | ELF1 | 0.256 |
| 2 | Sv_ELF1 | 0.238 | ELF1 | 0.323 | Sv_ACT | 0.227 | GAPDH1 | 0.283 |
| 3 | Sv_GAPDH1 | 0.238 | ACT | 0.332 | Sv_GAPDH2 | 0.247 | ACT | 0.321 |
| 4 | Sv_GAPDH2 | 0.274 | GAPDH2 | 0.338 | Sv_GAPDH1 | 0.271 | GAPDH2 | 0.470 |
| 5 | Sv_COG | 0.279 | ELF2 | 0.417 | Sv_COG | 0.416 | 18SrRNA | 0.623 |
| 6 | Sv_TUBA | 0.409 | COG | 0.571 | Sv_TUBA | 0.437 | ELF2 | 0.639 |
| 7 | Sv_ELF2 | 0.439 | TUBB | 0.829 | Sv_ELF2 | 0.475 | TUBB | 0.910 |
| 8 | Sv_TUBB | 0.863 | TUBA | 1.231 | Sv_18SrRNA | 0.508 | TUBA | 1.176 |
| 9 | Sv_CYP | 1.404 | CYP | 1.245 | Sv_TUBB | 1.042 | CYP | 1.217 |
| 10 | - *) | - | - | - | Sv_CYP | 1.315 | COG | 4.057 |

*) Sv_18SrRNA primer pair was not tested with oligo dT primed cDNA
